# The diagnostic and prognostic capability of artificial intelligence in spinal cord injury: A systematic review

**DOI:** 10.1016/j.bas.2025.104208

**Published:** 2025-02-05

**Authors:** Saran Singh Gill, Hariharan Subbiah Ponniah, Sho Giersztein, Rishi Miriyala Anantharaj, Srikar Reddy Namireddy, Joshua Killilea, DanieleS.C. Ramsay, Ahmed Salih, Ahkash Thavarajasingam, Daniel Scurtu, Dragan Jankovic, Salvatore Russo, Andreas Kramer, Santhosh G. Thavarajasingam

**Affiliations:** a Imperial Brain & Spine Initiative, Imperial College London, London, United Kingdom; b Faculty of Medicine, Imperial College London, London, United Kingdom; c Department of Neurosurgery, LMU University Hospital, LMU, Munich, Germany; d Department of Neurosurgery, Universitätsmedizin Mainz, Mainz, Germany; e Imperial College Healthcare NHS Trust, London, United Kingdom

**Keywords:** tSCI, Spinal cord injury, Spine, AI, Diagnosis, Prognosis

## Abstract

**Background:**

Artificial intelligence (AI) models have shown potential for diagnosing and prognosticating traumatic spinal cord injury (tSCI), but their clinical utility remains uncertain.

**Method:**

ology: The primary aim was to evaluate the performance of AI algorithms in diagnosing and prognosticating tSCI. Subsequent systematic searching of seven databases identified studies evaluating AI models. PROBAST and TRIPOD tools were used to assess the quality and reporting of included studies (PROSPERO: CRD42023464722). Fourteen studies, comprising 20 models and 280,817 pooled imaging datasets, were included. Analysis was conducted in line with the SWiM guidelines.

**Results:**

For prognostication, 11 studies predicted outcomes including AIS improvement (30%), mortality and ambulatory ability (20% each), and discharge or length of stay (10%). The mean AUC was 0.770 (range: 0.682–0.902), indicating moderate predictive performance. Diagnostic models utilising DTI, CT, and T2-weighted MRI with CNN-based segmentation achieved a weighted mean accuracy of 0.898 (range: 0.813–0.938), outperforming prognostic models.

**Conclusion:**

AI demonstrates strong diagnostic accuracy (mean accuracy: 0.898) and moderate prognostic capability (mean AUC: 0.770) for tSCI. However, the lack of standardised frameworks and external validation limits clinical applicability. Future models should integrate multimodal data, including imaging, patient characteristics, and clinician judgment, to improve utility and alignment with clinical practice.

## Highlights

• Diagnostic models show high accuracy (0.898) with advanced tools like MRI and CT scans.
• Prognostic models demonstrate moderate performance (AUC: 0.770) across various key outcomes.
• Models effectively predict AIS improvements, mortality, ambulation, and hospital stays.
• Challenges include overfitting, data bias, and lack of rigorous external model validation.
• Future AI must integrate multimodal data, ensure explainability, and undergo validation.

## 1 Introduction

Traumatic spinal cord injury (tSCI) involves trauma to the spinal cord, causing temporary or permanent motor, sensory, or autonomic deficits (Adegeest et al., 2024). This often results in conditions like paralysis and autonomic dysreflexia, significantly impairing quality of life (Adegeest et al., 2024; Konbaz et al., 2023; Kirshblum et al., 2011). TSCI, which affects either specific spinal regions, commonly cervical and thoracolumbar, or the entire spinal cord, account for up to 90% of spinal cord injuries, with incidence rates up to 906 cases per million globally (Adegeest et al., 2022; Ter Wengel et al., 2020; Barbiellini Amidei et al., 2022; Wang et al., 2022; Chen et al., 2021; Ullah et al., 2023). TSCI is a leading cause of disability in young people due to high-velocity trauma and falls (Chen et al., 2013; Wilson et al., 2023). It presents substantial challenges within healthcare, characterised by a poor prognosis and high mortality rates (Wilson et al., 2023; Higashi et al., 2018; Majdan et al., 2017; Hall et al., 2019).

To address these challenges, the World Society of Emergency Surgery (WSES) and the European Association of Neurosurgical Societies (EANS) developed a 17-point Delphi consensus to guide tSCI treatment, emphasising cross-specialty collaboration to improve outcomes of tSCI patients (Picetti et al., 2024). Current methods of tSCI diagnosis and prognostication involve time-intensive neurological consultations and imaging (Adegeest et al., 2024; Picetti et al., 2022; Wang et al., 2021; Badhiwala et al., 2021). They rely on multidisciplinary teams, rapid imaging, and assessment, though these methods can be time-consuming and risk inaccuracy (Adegeest et al., 2024; Picetti et al., 2022; Wang et al., 2021; Badhiwala et al., 2021). AI and ML present promising opportunities to streamline the assessment of tSCI and improve diagnostic accuracy (Nagendran et al., 2020; Masood et al., 2022; Facchinello et al., 2021).

Advancements in AI and ML for healthcare, driven by improvements in computational power, data availability, and deep learning algorithms, have enabled the development of versatile and complex models (LeCun et al., 2015). These models now have applications across various medical specialties (LeCun et al., 2015; Alowais et al., 2023; Kermany et al., 2018). Many AI models now automate clinical decisions with accuracy comparable to, or exceeding, that of healthcare professionals (HCPs) (Han et al., 2018; Nam et al., 2019; Gonzá et al., 2017; Shen et al., 2019; De et al., 2020; Hosny et al., 2018; Le et al., 2019). While few diagnostic models specifically target tSCI, AI's prognostic potential is strong, particularly using ML algorithms like XGBoost, random forests, and decision trees for predictive accuracy (Tay et al., 2014; McCoy et al., 2019; Jasim and Brindha, 2021; Chang et al., 2023; Karabacak and Margetis, 2023a; Dietz et al., 2022). However, research on AI based applications for tSCI in neurosurgery and neurotrauma remains limited.

To facilitate the adoption of newer diagnostic and prognostic methods for tSCIs, a robust quantitative and qualitative synthesis is needed. While current studies provide general insights into the application of AI in managing tSCI, this systematic review focuses on evaluating the performance and effectiveness of AI models in identifying and prognosticating tSCI (Dietz et al., 2022; Habibi et al., 2024; Maki et al., 2024; Tao et al., 2024; Graham et al., 2024). We aimed critically examine the inputs and outputs of existing AI models in the literature, to provide a the most comprehensive analysis of their capabilities within clinical environments.

## 2 Methodology

### 2.1 Search strategy and selection criteria

**Fig. 1:**
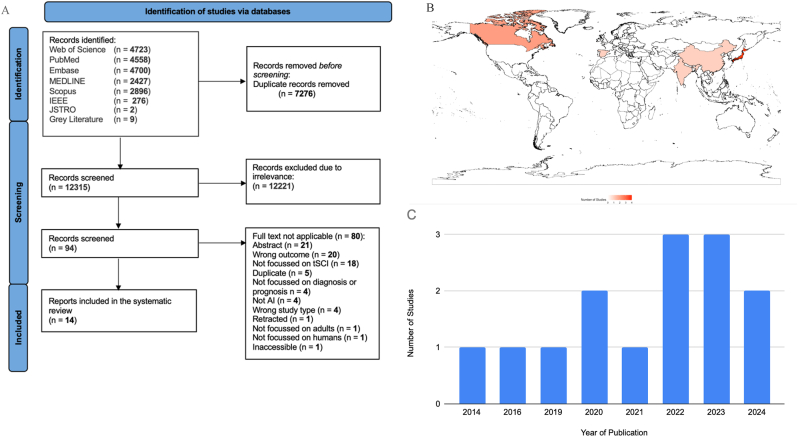
PRISMA diagram, World Map, Year of Publication A = PRISMA Diagram The PRISMA flowchart in Fig. 1A summarises the process of study identification, screening, and inclusion in this systematic review. A total of 12,315 records were identified from various databases, including Web of Science (4725), PubMed (4558), Embase (2700), Scopus (450), IEEE (Ullah et al., 2023), JSTOR (Chen et al., 2021), and Grey Literature (Ullah et al., 2023). After removing duplicates (7276 records), 5039 records were screened for relevance. Following the exclusion of 4945 irrelevant records, 94 full-text articles were assessed for eligibility. Of these, 80 articles were excluded for various reasons, such as lack of applicability (Namireddy et al., 2024), wrong population (Badhiwala et al., 2021), wrong outcomes (Higashi et al., 2018), and others, leaving 14 studies included in the systematic review. The chart adheres to PRISMA guidelines for systematic reviews. B = World Map Fig. 1B describes the global distribution of countries of publication of included studies, based on their frequency. Countries with higher frequencies are shaded in darker red, while those with lower frequencies are lighter. Non-listed countries are shown in white. The gradient in shading represents the number of occurrences, highlighting countries such as Japan, the United States, and Canada with the highest frequencies C = Year of Publication The graph in Fig. 1C shows a steady number of studies from 2014 to 2021, followed by an increase in 2022 and 2023, with a slight decline in 2024. Data points represent the total count of studies published in each respective year.

**Fig. 2:**
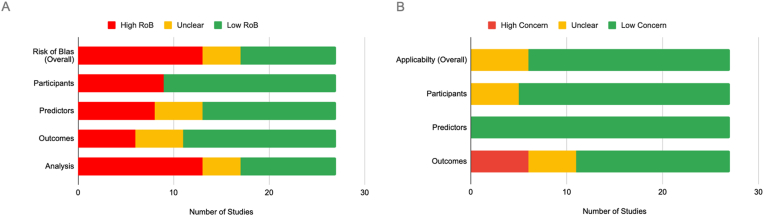
PROBAST Risk of Bias analysis. A = Risk of Bias domain This bar chart displays the number of studies rated as “High Risk,” “Unclear,” or “Low Risk” across different domains, including overall risk of bias, participants, predictors, outcomes, and analysis. A notable proportion of studies exhibit high or unclear risk, particularly in the “Analysis” and “Overall” categories. B = Applicability domain. This bar chart shows the number of studies with “High Concern,” “Unclear,” or “Low Concern” regarding applicability across the domains of participants, predictors, outcomes, and overall applicability. Most studies demonstrate low concern in these domains, though some uncertainty remains, particularly in the “Predictors” and “Outcomes” domains.

This systematic review was registered on PROSPERO (CRD42023464722) and was conducted in line with the Preferred Reporting Items for Systematic Reviews and Meta-Analyses (PRISMA) 2020 guidelines (Page et al., 2021). The completed PRISMA flowchart is shown in Fig. 1A. The literature search was conducted on the October 24, 2023, encompassing MEDLINE, Embase, Scopus, PubMed, JSTOR, IEEE, and the Cochrane Library. The complete search strategy can be found in Supplemental Digital Content 1: Supplementary Table S1. Four reviewers (SG, RMA, SN, JK) conducted the initial abstract screening using COVIDENCE. Studies using ML or AI for the diagnosis or prognostication tSCI, and met our inclusion criteria, were included. Our inclusion criteria included: original quantitative research, published in English, focused on AI for diagnosing or prognosticating tSCI in adults, and evaluated AI performance (Supplemental Digital Content 1: Supplementary Table S2). All included papers were then subject to a full text screen by five independent reviewers (SG, RMA, SN, JK, SSG). Any disagreements were resolved by consensus after discussion with a fifth reviewer (HSP) (see Fig. 2).

### 2.2 Data extractionfig2

Relevant data from each included study were manually extracted using COVIDENCE. A comprehensive list of extracted variables is presented in Supplemental Digital Content 1: Supplementary Table S6. In cases where data was missing, corresponding authors were contacted. Table 1, Table 2, Table 3 summarise key findings.

**Table 1:** Study characteristics.

Title	Author	Year	Country	Study type	Domain	Conclusion
Decision Tree Analysis Accurately Predicts Discharge Destination After Spinal Cord Injury Rehabilitation	Kato et al.	2024	Japan	Retrospective, single-centre study	Prognostication	Even during early stages of rehabilitation, it is possible to predict the discharge destination
Precision medicine for traumatic cervical spinal cord injuries: accessible and interpretable machine learning models to predict individualized in-hospital outcomes	Karabacak et al.	2024	USA	retrospective machine learning classification study	Prognostication	ML models showed strong predictive ability for in-hospital mortality and nonhome discharges, fair ability for prolonged LOS, but poor ability for prolonged ICU-LOS and major complications.
XGBoost, a Machine Learning Method, Predicts Neurological Recovery in Patients with Cervical Spinal Cord Injury	Inoue et al.	2020	Japan	Retrospective, single-centre study∗	Prognostication	The XGBoost model reliably predicted neurological changes in patients with cervical SCI
Machine Learning-based Prediction of Prolonged Intensive Care Unit Stay for Critical Patients with Spinal Cord Injury	Fan et al.	2022	China	Retrospective cohort study	Prognostication	Ensemble classifiers effectively predict prolonged ICU and hospital stays.
Determining the short-term neurological prognosis for acute cervical spinal cord injury using machine learning	Okimatsu et al.	2022	Japan	Retrospective cohort study	Prognostication	Predicting short-term neurological outcomes for acute cervical SCI using MRI and machine learning is feasible
Development of a machine learning algorithm for predicting in-hospital and 1-year mortality after traumatic spinal cord injury	Fallah et al.	2022	Canada	Retrospective review of a prospective cohort study	Prognostication	An ML based Spinal Cord Injury Risk Score (SCIRS) can predict in-hospital and 1-year mortality following tSCI more accurately than the ISS measure
Developing Artificial Neural Network Models to Predict Functioning One Year After Traumatic Spinal Cord Injury	Belliveau et al.	2016	USA	Retrospective analysis of data from the national, multicenter Spinal Cord Injury Model Systems (SCIMS) Database	Prognostication	Models for predicting ambulation status post tSCI were highly accurate, but require further prospective validation
Development of an unsupervised machine learning algorithm for the prognostication of walking ability in spinal cord injury patients	DeVries et al.	2020	Canada	Retrospective review of a prospective cohort study	Prognostication	No clinically significant differences were observed between the unsupervised ML algorithm using complete admission neurological data and previously validated standards.
Predictors of spinal trauma care and outcomes in a resource-constrained environment: a decision tree analysis of spinal trauma surgery and outcomes in Tanzania	Leidinger et al.	2023	Spain	Retrospective analysis of prospectively collected data	Prognostication	Operative intervention and functional improvement following acute spinal trauma were low and inconsistent.
Efficacy of a machine learning-based approach in predicting neurological prognosis of cervical spinal cord injury patients following urgent surgery within 24 h after injury	Shimizu et al.	2023	Japan	Retrospective consecutive cohort study	Prognostication	The ML models accurately predicted neurological outcomes six months post-injury in cervical SCI patients who underwent urgent surgery
Spinal Cord Injury AIS Predictions Using Machine Learning	Kapoor et al.	2023	USA	Retrospective NSCISC database analysis	Prognostication	AIS scores at admission, combined with demographic data, are highly predictive of neurological outcomes at hospital discharge for spinal cord injury patients
A machine learning approach for specification of spinal cord injuries using fractional anisotropy values obtained from diffusion tensor images	Tay et al.	2014	Republic of Korea	Retrospective study	Diagnosis	The ML based algorithm aids the diagnosis of SCI on DTI
Convolutional Neural Network-Based Automated Segmentation of the Spinal Cord and Contusion Injury: Deep Learning Biomarker Correlates of Motor Impairment in Acute Spinal Cord Injury	McCoy et al.	2019	USA	Retrospective, single-center study	Diagnosis	The segmentation tool performs better than currently available models. CNN improve algorithm performance and yields clinically relevant data for acute SCI patients
Spinal cord segmentation and injury detection using a Crow Search-Rider optimization algorithm	Jasim et al.	2021	India	Retrospective analysis	Diagnosis	The proposed model has been shown to be effective in SCI detection

This table describes the basic characteristics of all included studies, all of which develop an AI model to either diagnose or prognosticate SCI.

**Table 2:** Results and outcomes from all studies in the prognostication domain.

Study (year)	Input	Outcome	AI model	Sample size	AUC (95% CI)	Ground truth	Other performance measures
Kato et al. (2024)	SCIM total and subtotal scores, including self-care, respiration, sphincter management, and mobility	Discharge destination (home; not home)	CART	Training: 140 (66.7%) Testing: 70 (33.3%)	0.868 (0.755–0.960)	Retrospective database	Recall: 0.857 (0.759–0.955) Specificity: 0.810 (0.642–0.977) PPV: 0.913 (0.832–0.994) NPV: 0.708 (0.526–0.890)
Karabacak et al. (2023)	GCS (total), Age, GCS (Verbal) Pulse Oximetry, Respiratory assistance	In-hospital mortality (mortality; no mortality)	RF	Training: 42,997 (60%) Validation (3-time 5-fold cross-validation): 14,332 (20%) Testing: 14,332 (20%)	0.839 (0.816–0.848)	Retrospective database	Accuracy: 0.564 (0.556−0.572) Recall: 0.961 (0.958−0.964) Precision: 0.951 (0.947−0.955) AUPRC: 0.145 (0.139−0.151) Brier score: 0.028 (0.025−0.031)
	Age, Mechanism of Injury, Primary method of payment, Systolic blood pressure, Transport mode	Discharge destination (home; not home)	CB	Training: 40,399 (60%) Validation (3-time 5-fold cross-validation): 13,466 (20%) Testing: 13,466 (20%)	0.815 (0.803–0.818)	Retrospective database	Accuracy: 0.737 (0.73−0.744) Recall: 0.737 (0.73−0.744) Precision: 0.739 (0.732−0.746) AUPRC: 0.641 (0.633−0.649) Brier score: 0.177 (0.171−0.183)
	ACS Verification Level, systolic blood pressure, race, age, total GCS	Hospital LOS (>9 days; <9 days)	RF	Training: 46,070 (60%) Validation (3-time 5-fold cross-validation): 15,356 (20%) Testing: 15,356 (20%)	0.742 (0.721–0.742)	Retrospective database	Accuracy: 0.596 (0.588−0.604) Recall: 0.816 (0.81−0.822) Precision: 0.786 (0.78−0.792) AUPRC: 0.372 (0.364−0.38) Brier score: 0.128 (0.123−0.133)
	Systolic BP, ACS, Pulse oximetry, Pulse rate, primary method of payment	ICU LOS (>7 days; <7 days)	CB	Training: 15,969 (60%) Validation (3-time 5-fold cross-validation): 5323 (20%) Testing: 5323 (20%)	0.682 (0.657–0.696)	Retrospective database	Accuracy: 0.599 (0.586−0.612) Recall: 0.765 (0.754−0.776) Precision: 0.775 (0.764−0.786) AUPRC: 0.219 (0.208−0.23) Brier score: 0.13 (0.121−0.139)
Inoue et al. (2020)	Demogrpahics and neurological statis, mechanism of injury, treatment strategies, radiographic information, concomitant degenerative spine disease	AIS grade 6 months post-injury (D/E or A/B/C)	XGB	Training: 165 (8-fold cross-validation)	0.867	Retrospective database	Accuracy: 81.1%
Fan et al. (2022)	Mechanical ventilation, diagnosis, Red cell count, Haemoglobin, Magnesium	Hospital LOS (>14 days; <14 days)	EC	Training: 1012 (80%) Validation (3-time 5-fold cross-validation): 253 Testing: 253 (20%)	Validation: 0.815 Testing: 0.799	Retrospective database	Recall: 0.714 Specificity: 0.750 PPV: 0.481 NPV: 0.890
	Mechanical ventilation, diagnosis, LOS pre ICU, bicarbonate, chloride	ICU LOS (>7 days; <7 days)	EC	Training: 1279 (80%) Validation (3-time 5-fold cross-validation): 320 Testing: 320 (20%)	Validation: 0.864 Testing: 0.802	Retrospective database	Recall: 0.864 Specificity: 0.677 PPV: 0.479 NPV: 0.935
Okimatsu et al. (2022)	AIS probabilities, age and initial AIS at admission	AIS grade 1 month post-injury (A, B, C, D or E)	EC (CNN, RF, DL)	Training: 215 patients with 295 MR images (5-fold cross-validation)	–	Retrospective database	Accuracy: 0.714 Recall: 0.565 F1 score: 0.567 Precision: 0.59
Fallah et al. (2022)	age, AIS, NLI, Abbreviated Injury Scale scores, AOSpine injury morphology	Mortality 1 year post-injury (mortality; no mortality)	NN, DT	Training: 849 (validation: 10-fold cross-validation) Test: 396	Development: 0.84 Test: 0.86	Retrospective database	–
	age, AIS, NLI, Abbreviated Injury Scale scores, AOSpine injury morphology	In-hospital mortality (mortality; no mortality)	NN, DT	Training: 849 (validation: 10-fold cross-validation) Test: 396	Development: 0.87 Test: 0.85	Retrospective database	–
Belliveau et al. (2016)	–	Ambulate 150 ft 1 year post-discharge (yes; no)	ANN	Training: 2514 (80%) Cross-validation: 628 (20%)	0.8801 (0.8510–0.9092)	Retrospective database	Accuracy: 87.74% PLR: 8.59 NLR: 0.17
	–	Ambulate 1 street block 1 year post-discharge (yes; no)	ANN	Training: 2512 (80%) Cross-validation: 628 (20%)	0.8874 (0.8589–0.9159)	Retrospective database	Accuracy: 85.51% PLR: 5.46 NLR: 0.14
		Ambulate 1 flight of stairs 1 year post-discharge (yes; no)	ANN	Training: 2511 (80%) Cross-validation: 628 (20%)	0.9022 (0.8754–0.9290)	Retrospective database	Accuracy: 87.10% PLR: 7.57 NLR: 0.17
DeVries et al. (2020)	Age, AIS. L2-S1 motor function, light touch and pin prick	Walking ability 1 year post-injury (walk; no walk)	UML	Training: 862 (dataset re-sampling 2000 times)	0.89 (0.87–0.91)	Retrospective database	F1 score: 0.89 (0.87−0.91)
Leidinger et al. (2023)	–	In-hospital mortality (mortality; no mortality)	DT	Training: 284 (bootstrapping)	–	Retrospective database	Accuracy: 0.93 Recall: 0.96 Specificity: 0.71
	–	Improvement in AIS at discharge (>1 grade; no improvement)	DT	Training: 284 (bootstrapping)	–	Retrospective database	Accuracy: 0.34 Recall: 0.35 Specificity: 0.29
Shimizu et al. (2023)	AIS at admission, intramedullary haemorrhage, longitudinal T2WI hyperintensity, HbA1c, MSCC	Improvement in AIS 6 months post-injury (>1 grade; no improvement)	CB	Training: 101 (75%) Validation (5-fold cross-validation): 34 (25%)	0.90	Retrospective database	Accuracy: 0.837 Recall: 0.892 Precision: 0.852 F1 score: 0.872
	AIS at admission, intramedullary haemorrhage, longitudinal T2WI hyperintensity, HbA1c, MSCC	AIS grade 6 months post-injury (A, B, C, D, E)	CB	Training: 101 (75%) Validation (5-fold cross-validation): 34 (25%)	–	Retrospective database	Accuracy: 0.80 Recall: 0.572 Precision: 0.834 F1 score: 0.630
Kapoor et al. (2022)	AIS (A), AIS (D), AIS (C), Paraplegia, Tetraplegia	AIS grade (A, B, C, D, E)	RC	Training: 18,737 (90%) Testing: 2053 (10%)	–	Retrospective database	Train accuracy: 0.824 Test accuracy: 0.736

The input column contains the top 5 predictors for each study. SCIM: System for Cross-domain Identity Management; AIS: American Spinal Injury Association Impairment Scale; ANN: Artificial Neural Network; AUPRC: Area Under the Precision-Recall Curve; CART: Classification And Regression Tree; CB: CatBoost; CNN: Convolutional Neural Network; DL: Deep Learning; DT: Decision Tree; EC: Ensemble Classifier; ICU: Intensive Care Unit; LOS: Length Of Stay; NLR: Negative Likelihood Ratio; NN: Neural Network; PLR: Positive Likelihood Ratio; RC: Ridge Classifier; RF: Random Forest; XGB: XGBoost; USML: Unsupervised Machine Learning.

**Table 3:** Results and outcomes from all studies in the diagnostic domain.

Study (year)	Input	Output	AI model	Sample size (no. images)	No. Images	AUC (95% CI)	Ground truth	Other performance measures
Tay et al. (2014)	DTI	Spinal cord injury (injured; normal)	SVM, KNN	14 (164)	Training: 164 (3-fold cross validation)	–	Experts at University	Accuracy: 0.938 Recall: 0.952 Specificity: 0.912
McCoy et al. (2019)	T2W1 MRI	Volume of spinal contusion	CNN	47 (1880)	Training: 1120 (60%) Validation: 200 (10%) Testing: 560 (30%)	–	Neuroradiology fellows	Dice coefficient: 0.93
Jasim et al. (2021)	CT lumbar spine with VCF	Spinal cord injury	CS-ROA DCNN	30 (Hosny et al., 2018)	Training: 15 (50%) Validation: 15 (50%)	–	Retrospective database	Accuracy: 0.8128 Sensitivity: 0.8233 Specificity: 0.7568
	CT lumbar spine with VCF	Spinal cord injury	CS-ROA DCNN	30 (Hosny et al., 2018)	Training: 18 (60%) Validation: 12 (40%)	–	Retrospective database	Accuracy: 0.8204 Sensitivity: 0.8364 Specificity 0.8378
	CT lumbar spine with VCF	Spinal cord injury	CS-ROA DCNN	30 (Hosny et al., 2018)	Training: 21 (70%) Validation: 9 (30%)	–	Retrospective database	Accuracy: 0.8672 Sensitivity: 0.8703 Specificity 0.8919
	CT lumbar spine with VCF	Spinal cord injury	CS-ROA DCNN	30 (Hosny et al., 2018)	Training: 24 (80%) Validation: 6 (20%)	–	Retrospective database	Accuracy: 0.88 Sensitivity: 0.8745 Specificity 0.8964
	CT lumbar spine with VCF	Spinal cord injury	CS-ROA DCNN	Hosny et al. (2018)	Training: 27 (90%) Validation: 3 (10%)	–	Retrospective database	Accuracy: 0.886 Sensitivity: 0.8964 Specificity 0.8986

VCF: Vertebral compression fracture; CNN: Convolutional Neural Network; DL: Deep Learning; DTI: Diffusion Tensor Imaging; KNN: K-Nearest Neighbour; SVM: Support Vector Machine; T2W1 MRI: T2 Weighted Magnetic Resonance Image, CS-ROA DCNN: Crow search-Rider Optimization-based DCNN.

### 2.3 Critical appraisal

Two independent reviewers assessed the risk of bias for each included study using the Prediction model Risk Of Bias Assessment Tool (PROBAST), evaluating potential biases in four domains: participant selection, predictors, outcomes, and data analysis (Collins et al., 2021; Wolff et al., 2019). Any disagreements in risk assessment were resolved by a third reviewer. Additionally, adherence to the Transparent Reporting of a multivariable prediction model for Individual Prognosis Or Diagnosis (TRIPOD) guidelines was evaluated for each study, to ensure transparency and completeness in reporting (Collins et al., 2015).

### 2.4 Data analysis, qualitative synthesis and reporting

**Fig. 3:**
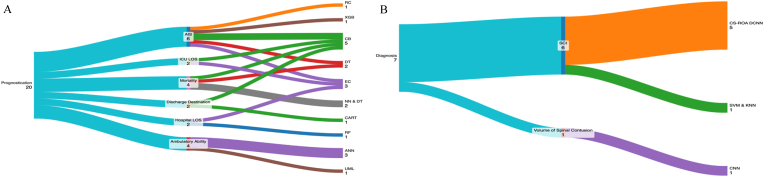
Sankey Diagram. A: This diagram displays the type of model used by each outcome in the prognostication arm of this study CatBoost (CB) was the most commonly used model, appearing in 20% (4/20) of the included models. Artificial Neural Networks (ANN) also appeared in 15% (3/20) of the models, as did Ensemble Classifiers (EC), which included one ensemble combining Convolutional Neural Networks (CNN), Random Forest (RF), and Deep Learning (DL) (Campbell et al., 2020; Kato et al., 2024; Inoue et al., 2020). Random Forest (RF) models alone were used in 10% (2/20) of the models, as were Decision Trees (DT) models alone. A combination of Neural Networks (NN) and Decision Trees (DT) was utilised in 10% (2/20) of the models (Karabacak and Margetis, 2023b). Classification and Regression Trees (CART), XGBoost (XGB), Unsupervised Machine Learning (UML), and Ridge Classifier (RC) were each used in 5% (1/20) of the models.B: This diagram displays the type of model used by each outcome in the diagnosis arm of this study Six of the seven models were based on neural networks. Specifically, five models utilised a Crow Search-Rider Optimization-based Deep Convolutional Neural Network (CS-ROA DCNN), with one model based exclusively on Convolutional Neural Networks (CNN). The remaining model employed a combination of Support Vector Machines (SVM) and k-Nearest Neighbors (KNN). These models used a total of 2194 images for testing and validation. Accuracy and specificity were the most commonly reported metrics, reported for 86% (6/7) of models followed by sensitivity (5/7, 71%), dice and recall (1/7, 14%). AIS: American Spinal Injury Association Impairment Scale; ANN: Artificial Neural Network; CART: Classification And Regression Tree; CB: CatBoost; CNN: Convolutional Neural Network; DL: Deep Learning; DT: Decision Tree; EC: Ensemble Classifier; ICU: Intensive Care Unit; LOS: Length Of Stay; ML: Machine Learning; NN: Neural Network; RC: Ridge Classifier; RF: Random Forest; XGB: XGBoost; USML: Unsupervised Machine Learning; KNN: K-Nearest Neighbour; SVM: Support Vector Machine; CS-ROA DCNN: Crow Search-Rider Optimization-based DCNN.

**Fig. 4:**
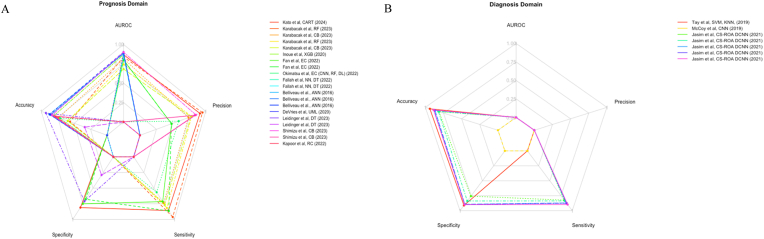
A = Radar Chart of the Prognosis Domain AI algorithms performance metrics. Fig. 4A illustrates the performance metrics of various AI algorithms, as reported by each article's authors, in the prognosis domain. The radar chart specifically showcases 5 key metrics: Area Under the Curve (AUC), Accuracy, Precision, Specificity, and Sensitivity. Each vertex of the radar chart corresponds to one of these metrics, scaled from 0 to 1, where 1 denotes optimal performance. For the sake of graphical illustration, a metric value of 0 is equivalent to “NR” (Not Reported), indicating that the specific performance metric was not disclosed in the respective study. A mean of testing and training data was used where both were availableA = Radar Chart of the Diagnosis Domain AI algorithms performance metrics. Fig. 4B illustrates the performance metrics of various AI algorithms, as reported by each article's authors, in the diagnosis domain. The radar chart specifically showcases 5 key metrics: Area Under the Curve (AUC), Accuracy, Precision, Specificity, and Sensitivity. Each vertex of the radar chart corresponds to one of these metrics, scaled from 0 to 1, where 1 denotes optimal performance. For the sake of graphical illustration, a metric value of 0 is equivalent to “NR” (Not Reported), indicating that the specific performance metric was not disclosed in the respective study. A mean of testing and training data was used where both were available ANN: Artificial Neural Network; CART: Classification And Regression Tree; CB: CatBoost; CNN: Convolutional Neural Network; DL: Deep Learning; DT: Decision Tree; EC: Ensemble Classifier; ML: Machine Learning; NN: Neural Network; RC: Ridge Classifier; RF: Random Forest; XGB: XGBoost; USML: Unsupervised Machine Learning; KNN: K-Nearest Neighbour; SVM: Support Vector Machine; CS-ROA DCNN: Crow Search-Rider Optimization-based DCNN.

Due to the heterogeneity of methodologies, including differences in model architecture, predictive features, and outcome measures, a meta-analysis was not feasible. Instead, a qualitative synthesis was conducted, following the Synthesis Without Meta-analysis (SWiM) guidelines (Campbell et al., 2020). Data interpretation was enhanced through visual representations created using Google Sheets and R statistical packages, with Radar and Sankey diagrams (Fig. 3, Fig. 4).

## 3 Results

A total of 12,315 studies were screened. From these, 94 full texts were assessed using our inclusion criteria. A total of 14 studies, and a pooled total of 283,046 images in the diagnostic and prognostic domains, respectively, were included in this systematic review (Tay et al., 2014; McCoy et al., 2019; Jasim and Brindha, 2021; Kato et al., 2024; Karabacak and Margetis, 2023b; Inoue et al., 2020; Fan et al., 2022; Okimatsu et al., 2022; Fallah et al., 2022; Belliveau et al., 2016; DeVries et al., 2020; Leidinger et al., 2023; Shimizu et al., 2023; Kapoor and Xu, 2023). AI models predicted prognosis in 11 studies (79%), and diagnosis in 3 studies (21%). Characteristics of all included studies are found in Fig. 1B and C and Table 1, Table 2, Table 3, describing the diagnostic and prognostic arms of this study, respectively.

### 3.1 Prognostication of tSCI

This domain of the study focussed on the use of AI in the prognostication of tSCI, comprising of 20 models (Kato et al., 2024; Karabacak and Margetis, 2023b; Inoue et al., 2020; Fan et al., 2022; Okimatsu et al., 2022; Fallah et al., 2022; Belliveau et al., 2016; DeVries et al., 2020; Leidinger et al., 2023; Shimizu et al., 2023; Kapoor and Xu, 2023). 280,852 images were used to train these models, with the individual sample sizes ranging from 135 to 72,132.40% (8/20) of models were composed of less than 1000 samples. AUC was the most reported outcome, with a weighted mean of 0.770, reported in 75% of models (15/20), followed by accuracy (14/20) and recall (12/20) respectively (Table 1, Table 2, Fig. 3a). The system used by each model is shown in Fig. 4a. Improved American Spinal Injury Association Abbreviated Injury Scale (AIS) was the most predicted outcome, appearing in 30% (6/20) of the included models. Mortality and post-injury ambulatory ability were each predicted in 20% (4/20) of the models, followed by discharge destination, ICU and hospital lengths of stay in 10% (2/20) of the models (Fig. 3a).

### 3.2 Diagnosis of tSCI

This domain of the study focussed on the use of AI in the detection of spinal cord injuries and is based seven models and trained on 2194 images (Tay et al., 2014; McCoy et al., 2019; Jasim and Brindha, 2021). All studies were retrospective, and the ground truth was based on reports from neuroradiology fellows, a retrospective database, and university experts, respectively (Table 1, Table 3, Fig. 3b). A mean weighted average of all included models was 0.898.6 models (85.7%) diagnosed SCI directly using the binary outcomes of ‘SCI or ‘no SCI’ using DTI and CT scans (Tay et al., 2014; Jasim and Brindha, 2021). The remaining model (14.3%) diagnosed the volume of spinal contusion in SCI from T2W1 MRI scans (McCoy et al., 2019). The system used by each model is shown in Fig. 3b. Accuracy and specificity were the most reported outcomes, reported in 85.7% (6/7) of all included models.

### 3.3 Data input for AI models

#### 3.3.1 Age and demographics

Five models identified age as the most critical predictive factor. Inoue et al. included “demographics,” encompassing age, as the primary predictor for AIS grade six months post-tSCI (Inoue et al., 2020). Fallah et al. found age to be the strongest predictor of both one year and in hospital mortality post-tSCI (Fallah et al., 2022), while DeVries et al. determined it as the best predictor of ambulatory ability one year post-tSCI (DeVries et al., 2020). Karabacak et al. calculated age to have the highest SHapley Additive exPlanations (SHAP)value, a measure quantifying the importance of input features in a ML model, (+0.25) for non-home discharge, far exceeding the next highest factor (0.06) (Karabacak and Margetis, 2023b).

#### 3.3.2 AIS

AIS was the most significant predictive feature in four models. Kapoor et al. found AIS grades A, D, and C to be the top three features for predicting final AIS grade (Kapoor and Xu, 2023). Shimizu et al. used AIS at admission to predict AIS improvement six months post-tSCI (Shimizu et al., 2023). Similarly, Okimatsu et al. ranked AIS probabilities, and AIS at admission, as the first and third most important features for predicting AIS improvements one-month post-tSCI (Okimatsu et al., 2022).

#### 3.3.3 Images

Three studies (7 models) employed imaging to diagnose SCI. Jasim et al. used segmented and localised L1-5 CT scans (Jasim and Brindha, 2021), while both McCoy et al. and Tay et al. utilised MRI (Tay et al., 2014; McCoy et al., 2019). McCoy et al. used a 3T MRI of the cervical vertebrae with axial and sagittal T2 fast spin-echo sequences (McCoy et al., 2019). Whereas Tay et al. incorporated axial T1 and T2 scans with diffusion tensor imaging (DTI), derived from diffusion tensor measures to train their model (Tay et al., 2014).

##### 3.3.3.1 Mechanical ventilation

Two models highlighted mechanical ventilation as the most important feature in their ensemble classifiers. Decision curve analysis and feature permutation assessments showed scores of 0.065 and 0.035 for predicting ICU and hospital length of stay, respectively (Fan et al., 2022).

#### 3.3.4 Disability measures

Two models found that disability was the most important feature in their programming. Kato et al. found that the Spinal Cord Independence Measure (SCIM) is the best predictor of discharge location (Kato et al., 2024). Yet, Karabacak et al. found that the total score on the Glasgow Coma Scale (GCS), followed closely by the verbal component of the GCS, are the primary predictors of in hospital mortality for tSCI patients (Karabacak and Margetis, 2023b).

#### 3.3.5 Miscellaneous

Karabacak et al. found that both ACS verification levels, representing a hospital's ability to deal with trauma, and a patient's systolic blood pressure are the best predictors of length of stay in the hospital and ICU, respectively (Karabacak and Margetis, 2023b).

### 3.4 Algorithms and validation of AI model

#### 3.4.1 Machine Learning algorithms and performance

Convolutional Neural Networks (CNNs) achieved the highest mean accuracy (86.78%) and AUROC (0.8899) across three models for prognosticating tSCIs. XGBoost (XGB) followed with 81.1% accuracy and an AUROC of 0.867 from one model. Ridge Classifiers (RC) ranked third with 78% accuracy, though AUROC was unreported. CatBoost (CB) recorded 74.33% accuracy and a mean AUROC of 0.799 across four models, and Ensemble Classifiers (ECs) delivered 71.4% accuracy and 0.828 AUROC over five models. These ECs incorporating Neural Networks (NNs), Decision Trees (DTs), Random Forests (RFs), and Deep Learning (DL). Decision Trees (DTs) showed 63.5 accuracy without AUROC data from one model. RFs reported 58 accuracy and 0.791 AUROC from two models. CART lacked accuracy data but achieved an AUROC of 0.868 with one model, while UML reported a high AUROC of 0.89 without accuracy, based on one model. Leidinger et al.’s DT excelled in mortality prediction with 93% accuracy and 96% sensitivity, making it the best prognostic model (Leidinger et al., 2023). However, its accuracy and sensitivity for predicting AIS improvements (34% and 35%, respectively) were the lowest in this study (Leidinger et al., 2023). In the diagnostic domain, Tay et al.’s EC, outperformed other CNNs with mean accuracies of 93.8% and 85.33%, respectively (Tay et al., 2014). Their model combined SVM and KNN (Tay et al., 2014). Tay et al.’s KNN model, optimised with two features, outperformed their SVM model (93.8% vs. 93.3% accuracy), using eight features (Tay et al., 2014). Despite focusing only on C4-6 SCI, Tay et al.’s model was the top performer in this domain (Tay et al., 2014).(Fig. 4)

#### 3.4.2 Validation methods

The majority of studies used cross validation techniques, between 5 and 10 cross-folds. Only Tay et al. and Jasim et al. used k-fold cross-validation as their approach (Tay et al., 2014; Jasim and Brindha, 2021). Four studies did not specify their validation methods (Kato et al., 2024; Karabacak and Margetis, 2023b; Leidinger et al., 2023; Kapoor and Xu, 2023), and DeVries et al. compared their model to a previously validated model, but did not perform formal validation (DeVries et al., 2020).

#### 3.4.3 Output and outcome measures

The American Spinal Injury Association (AIS) scale was the most common target for tSCI prognostication, with models achieving 71.37% mean accuracy and an AUC of 0.8835 for binary AIS improvement predictions (Table 3, Table 4). Ambulatory ability predictions showed mean AUCs of 0.890 and accuracies of 86.78%, demonstrating strong reliability for post-discharge mobility forecasts. Mortality models achieved a mean accuracy of 74.7%, and AUC of 0.841, using predictors like age and Glasgow Coma Scale (GCS) scores. Models for prolonged hospital and ICU stays also performed well, with mean AUCs of 0.771 and 0.742, respectively. For the diagnosis of SCI, detection was performed with a mean accuracy of 86.74%, while the volume of spinal cord contusion was diagnosed with a DICE coefficient of 0.93 (see Table 5).

**Table 4:** PROBAST.

Study		Risk of Bias				Applicability			Overall	
Author (year)	AI Model	Participants	Predictors	Outcomes	Analysis	Participants	Predictors	Outcomes	ROB	Applicability
Kato et al. (2023)	CART	L	L	L	H	L	L	L	H	L
Karabacak et al. (2023)	RF	L	L	L	L	L	L	L	L	L
	CB	L	L	L	L	L	L	L	L	L
	RF	L	L	L	L	L	L	L	L	L
	CB	L	L	L	L	L	L	L	L	L
Inoue et al. (2020)	XGB	L	U	L	L	L	L	L	L	L
Fan et al. (2021)	EC	L	U	H	U	L	L	H	U	U
	EC	L	U	H	U	L	L	H	U	U
Okimatsu et al. (2022)	EC (CNN, RF, DL)	L	H	H	U	L	L	H	H	U
Fallah et al. (2022)	NN, DT	L	L	L	H	L	L	L	L	L
	NN, DT	L	L	L	H	L	L	L	L	L
Belliveau et al. (2016)	ANN	L	H	H	L	L	L	H	H	U
	ANN	L	H	H	L	L	L	H	H	U
	ANN	L	H	H	L	L	L	H	H	U
DeVries et al. (2020)	UML	L	H	L	U	L	L	L	H	L
Leidinger et al. (2023)	DT	H	L	L	H	L	L	L	L	L
	DT	H	U	L	H	L	L	L	U	L
Shimizu et al. (2023)	CB	L	H	L	L	L	L	L	L	L
	CB	L	H	L	L	L	L	L	L	L
Kapoor et al. (2022)	RC	L	H	L	H	L	L	L	H	L
Tay et al. (2014)	SVM, KNN	H	L	L	H	L	L	L	U	L
McCoy et al. (2019)	CNN	H	U	L	H	L	L	L	H	L
Jasim et al. (2021)	CS-ROA DCNN (50% Training)	H	L	U	H	U	L	U	H	L
	CS-ROA DCNN (60% Training)	H	L	U	H	U	L	U	H	L
	CS-ROA DCNN (70% Training)	H	L	U	H	U	L	U	H	L
	CS-ROA DCNN (80% Training)	H	L	U	H	U	L	U	H	L
	CS-ROA DCNN (90% Training)	H	L	U	H	U	L	U	H	L

Table 4 describes the PROBAST analysis for each subsection. H=High; U=Unclear; L = Low; ROB=Risk of Bias; ANN: Artificial Neural Network; CART: Classification And Regression Tree; CB: CatBoost; CNN: Convolutional Neural Network; DL: Deep Learning; DT: Decision Tree; EC: Ensemble Classifier; ML: Machine Learning; NN: Neural Network; RC: Ridge Classifier; RF: Random Forest; XGB: XGBoost; USML: Unsupervised Machine Learning; KNN: K-Nearest Neighbour; SVM: Support Vector Machine; CS-ROA DCNN: Crow Search-Rider Optimization-based DCNN.

**Table 5:** – Mean AUROC and accuracy for each domain.

Outcome Group	Mean AUROC	Mean Accuracy
AIS	0.8835	71.37%
Ambulation	0.8899	86.78%
Discharge	0.8415	73.70%
Hospital LOS	0.7705	59.60%
ICU LOS	0.7420	59.90%
Mortality	0.8497	74.70%
SCI	N/A	86.74%

This table depicts the mean AUROC to 4 decimal places, and accuracy to 2 decimal places, of each outcome domain. Contusion volume was excluded due to no data on AUROC or accuracy.

### 3.5 Appraisal of AI models and studies

#### 3.5.1 TRIPOD assessment

TRIPOD adherence, excluding items considered “not applicable”, ranged from 0% to 100%, with a mean adherence of 74.30%. Nine items had total (100%) adherence: 1, 3a, 3b, 7a, 10b, 10d, 18, 19b, and 20. Items 10e, 11, and 17 were considered “not applicable” to the majority of the articles, reflecting the focus on model development without external validation.

#### 3.5.2 PROBAST assessment

After a thorough evaluation, 48% of the studies included in this review were considered to have a high risk of bias (ROB), primarily due to issues in the analysis domain. A smaller proportion (15%) were classified as having an unclear ROB, while 37% were rated as having a low ROB. Outcome applicability was a high concern in 22% of the articles, while participant and predictor applicability were consistently rated as low risk (100%). Overall, this resulted in a low concern for applicability in 78% of the studies, with the remaining 22% showing unclear applicability (Table 4).

## 4 Discussion

This systematic review, without a meta-analysis, synthesises the growing body of literature on the potential and performance of AI in diagnosing and prognosticating tSCI. It encompasses 14 studies and includes data from 27 models. Several studies highlighted models with high discriminatory power and robust performance, particularly in predicting ambulatory ability, in-hospital mortality, and tSCI diagnosis using various imaging modalities, such as DTI, MRI, and CT (Tay et al., 2014; McCoy et al., 2019; Jasim and Brindha, 2021; Kato et al., 2024; Karabacak and Margetis, 2023b; Inoue et al., 2020; Fan et al., 2022; Okimatsu et al., 2022; Fallah et al., 2022; Belliveau et al., 2016; DeVries et al., 2020; Leidinger et al., 2023; Shimizu et al., 2023; Kapoor and Xu, 2023).Our findings suggest that AI shows significant promise in improving tSCI risk stratification and supporting clinical decision-making. Future advancements in AI should aim to integrate the predictive precision of traditional ML with the diagnostic accuracy of deep learning to enhance the overall assessment and management of tSCI.

The high specificity and AUROC reported in studies, such as those by Karabacak et al. and Kapoor et al., underscore the potential of AI as an adjunct to radiologists in interpreting scans for tSCI patients (Karabacak and Margetis, 2023b; Kapoor and Xu, 2023). By working alongside radiologists, AI can help address the clinical need for rapid management of tSCI, reducing the reliance on a second radiologist to review complex scans and significantly improving the speed and efficiency of scan interpretation (Picetti et al., 2024; Mahmoudi et al., 2021; Hosman et al., 2023; Mora-Boga et al., 2023). While no studies have comprehensively compared the time efficiency of radiologists with and without AI assistance in diagnosing tSCI, broader research by Meng F et al. suggests that AI can significantly reduce diagnostic time in other contexts, such as COVID-19 (p<0.01) (Meng et al., 2023). In their study, using 780 CT images from a multinational dataset consisting of COVID-19 or community-acquired pneumonia positive scans, AI assistance saved an average of 9.3 min per diagnosis (p<0.01) (Meng et al., 2023). Therefore, this warrants further investigation specifically for tSCI (Meng et al., 2023). Furthermore, models should be trained across a diverse demographic of patients, aiming to avoid the ‘black box’ effect, wherein AI decision-making lacks transparency, and focus on explainable AI (XAI) (Wadden, 2021; Javed et al., 2023). Future models should prioritise XAI to provide a clear rationale or justification for their decisions, enhancing both their utility and validity in clinical settings (Ali et al., 2023). Methods such as SHAP and LIME (Local Interpretable Model-Agnostic Explanations) can pinpoint the features that most influenced a prediction, including patient demographics, lab results, or imaging findings (Aldughayfiq et al., 2023). By offering this level of transparency, XAI enables clinicians to validate a model's reasoning against their clinical expertise, assess its reliability across different scenarios, and identify potential biases. Ultimately, XAI fosters greater confidence in AI models and could allow for its integration into clinical workflows.

Our study demonstrated that AI offers varying levels of clinical utility. For instance, Kato et al. showed that discharge destination can be accurately predicted early in rehabilitation, enabling tailored care plans and improved discharge outcomes (Kato et al., 2024). Accurate predictions are vital, as they may necessitate modifications to patients’ houses or additional support, including transitions into care facilities (Abad et al., 2021; Badawi and Breslow, 2012; Zimmerman et al., 1994). Additionally, leveraging these predictions to prioritise pre-discharge education can address common gaps in patient knowledge, ensuring that discharge planning for tSCI patients can be thorough and patient focussed (New, 2015; Simpson et al., 2012). The rates of readmission for SCI patients are known to be high, ranging from 28 to 45% within the first year (Hiremath et al., 2021). While pre-discharge education has not been formally assessed in tSCI patients, it has proven effective in improving post-discharge outcomes in other fields. Oh et al., found that adequate pre-discharge education can significantly reduce readmission rates and complications in heart failure patients (Oh et al., 2023). If similar educational strategies were applied to tSCI patients and were tailored according to patient specific information using AI models, they could potentially improve outcomes by addressing individual care needs, enhancing self-management, and reducing the likelihood of post-discharge complications. Similarly, predicting AIS grades was a frequent focus in the review. While AIS is a validated and widely used assessment tool for SCI, it is not without limitations (Marino et al., 2008; Roberts et al., 2017). It does not fully capture injury severity or symptoms like pain or spasticity, whilst some components of the score, such as the bulbocavernosus reflex, are challenging to measure in clinical settings (Marino et al., 2008; Roberts et al., 2017; Hunt and McQuillan, 2023). Despite the continued debate around the minimal clinically important difference (MCID) for AIS, some authors view an improvement of one AIS grade as significant (Marino et al., 2008; van Middendorp et al., 2009; Samdani et al., 2011). As such, the outcomes and predictive features determined by the included algorithms, although significant, may not offer meaningful clinical insights for tSCI patients.

Applying predictive models for tSCI in clinical practice allows for the incorporation of more widely used and validated clinical data into model training, improving predictive accuracy and providing evidence-based monitoring parameters (Shimizu et al., 2023). Namely, Shimizu et al. presented a ML model to predict neurological prognosis post cervical SCI in clinical practice (Shimizu et al., 2023). They used readily available parameters like HbA1c, alcohol intake, and MRI features to predict AIS grades six months post-SCI (Shimizu et al., 2023). These findings demonstrate clinical utility and could reshape the initial workup for suspected tSCI, potentially improving outcomes and prognoses by using data that may already be present in patients’ notes. This underscores the need for a critical appraisal of current practices, including evaluations of existing guidelines and predictive models, to identify novel clinical features that could enhance clinician performance and offer novel variables for AI models for tSCI to incorporate and ultimately improve care for tSCI patients.

Performance comparisons between ML models and clinicians or existing prognostication algorithms, such as regression models, were limited. Overall, the discriminatory ability of ML models was comparable to that of logistic regression (LR) when evaluated on the same sample, with median AUCs of 0.88 (0.87–0.89) for ML models and 0.87 (0.87–0.88) for LR models. Notably, we observed superior AUCs for LR when trained and validated on larger sample sizes. Poor data quality, bias, overfitting, and limited features may explain ML's disadvantages in these settings (Rajput et al., 2023). Christodoulo et al. suggest that there is no significant difference between the performance of ML and LR based models, with 0 (95%CI: −0.18 – 0.18) difference in AUC. They also suggest that any observed differences often result from methodological flaws, including homogeneity in reporting transparency (Christodoulou et al., 2019). Despite this, ML is often regarded as more accurate and powerful than LR, largely due to its ability to automatically determine the relative importance of predictive features (Cruz and Wishart, 2007; Panesar et al., 2019). Additionally, ML is less restrictive and better equipped to address non-linear relationships, making it more suitable for predictive modelling in large datasets, such as those required to draw valid conclusions for tSCI, than LR (Panesar et al., 2019; Lee et al., 2018; Khan et al., 2019).

DeVries et al. highlighted how dataset imbalance can skew AUC, with ML models producing higher false-negative rates despite having similar AUCs to logistic regression (DeVries et al., 2020). To address this, future research should prioritise metrics like the F1 score, which are more robust against imbalanced datasets and provide more reliable performance evaluations (Seo et al., 2021). AUC, while widely used, is prone to over-optimism in the presence of class imbalance, making its outputs less generalisable to the diverse presentations seen in clinical practice (Seo et al., 2021; Buda et al., 2018). Similarly, the lack of external validation also limits conclusions about ML's superiority over traditional methods, a recurring issue in novel ML studies (Siontis et al., 2015; Pauling et al., 2024; Namireddy et al., 2024). Large, multicentred datasets focused on tSCI imaging and clinical data are needed to enable rigorous validation and standardisation of future models. However, overcoming challenges such as the initial cost and server space required for a large number of high-resolution scans, issues of patient consent, compliance with General Data Protection Regulation (GDPR) laws, and the logistical complexities of coordinating the creation of a large dataset are essential to achieve this (Pauling et al., 2024; Chico, 2018). Overfitting was a common issue, with seven studies addressing it explicitly (Tay et al., 2014; McCoy et al., 2019; Karabacak and Margetis, 2023b; Inoue et al., 2020; Okimatsu et al., 2022; Belliveau et al., 2016; Shimizu et al., 2023). Many studies had events per variable (EPVs) below 10, indicating that small sample sizes with numerous predictors risked overfitting by including spurious predictors (Wolff et al., 2019). Most studies poorly handled missing data, often failing to report its presence, extent, or management. PROBAST guidelines recommend multiple imputation to mitigate selection bias and maintain data integrity (Wolff et al., 2019; Janssen et al., 2010). However, high levels of missing data increase bias risk, with the acceptable threshold for imputation or exclusion remaining unclear (Wolff et al., 2019; Rizvi et al.)^.^

Future model development should apply these principles to enhance clinical utility for HCPs managing tSCIs. All included studies were retrospective, using pre-existing databases with limited features and lacking specific protocols, likely contributing to disparities in model performance due to unaccounted confounders, database granularity, and poor handling of missing data. The absence of quality control for datasets further complicates the reliability of model training (Liu et al., 2019). While most studies conducted internal validation, inconsistencies in defining and distinguishing between internal and external validation were common. True external validation, critical for assessing model transportability, was often unclear, emphasising the need for out-of-sample testing (Van Calster et al., 2023; Steyerberg and Harrell, 2016).

Furthermore, clinical prediction model guidelines recommend resampling techniques such as cross-validation and bootstrapping for internal validation (Collins et al., 2021; Wolff et al., 2019). However, some studies used simpler methods, such as random data splits into testing and training samples, that inadequately address optimism since models aren't trained on all available data (Collins et al., 2021; Wolff et al., 2019; Christodoulou et al., 2019). Reporting of model performance was inconsistent, with many omitting calibration and clinical utility assessments, including the 95% confidence intervals, despite PROBAST and TRIPOD guidelines, limiting the clinical applicability of their findings (Collins et al., 2021; Wolff et al., 2019).

The study's strengths include a comprehensive literature search following PROBAST, TRIPOD, and PRISMA guidelines. Despite rigorous methods, some limitations remain. Individual study outcomes were treated as separate models, with limited information on missing data handling and analysis procedures. Sample sizes varied significantly, with some studies testing models on as few as 34 data points. Heterogeneity among studies and diverse AI models with unique methodologies prevented the conduction of a meta-analysis, warranting cautious interpretation of findings and raising concerns about the adequacy of evidence to support the routine clinical use of these AI models. This variability reflects ongoing challenges in standardising and regulating AI methodologies in healthcare complicating comparisons and limiting broad conclusions (Namireddy et al., 2024; Marwaha and Kvedar, 2022; Cimpeanu et al., 2022). Thereby, slowing the process of updating best practices for managing conditions such as tSCI, ultimately impacting tSCI patient care (Namireddy et al., 2024; Cimpeanu et al., 2022).

This review highlights the potential of AI and ML in managing tSCIs, particularly in predicting outcomes like ambulatory ability, mortality, and injury detection. Diagnostic models showed strong performance with a weighted accuracy of 0.898, while prognostic models had more variability, with a weighted mean AUC of 0.770. Some prognostic studies approached AUCs near 0.9, but many fell closer to 0.7, indicating room for improvement. Methodological issues like overfitting, inconsistent data handling, and insufficient validation, along with high development costs, hinder immediate clinical adoption. Future research should address these methodological shortcomings, improve model validation, and assess cost-effectiveness to better establish the role of AI in tSCI care and ensure its integration into clinical practice.

## Previous presentation

No.

## Data availability statement

All relevant data supporting the findings of this study can be accessed within the Supplementary Digital Content, and tables, attached to the article.

## Additional information

The authors declare that no funds, grants, or other support were received during the preparation of this manuscript. The authors have no relevant financial or non-financial interests to disclose. All data and materials as well as software application support their published claims and comply with field standards. Consent to publish has been received from all participants.

## Funding

None.

## Declaration of competing interest

The authors declare that they have no known competing financial interests or personal relationships that could have appeared to influence the work reported in this paper.
